# Comparison of Screening Colonoscopy Rates After Positive Noninvasive Testing for Colorectal Cancer in States With and Without Cost-Sharing

**DOI:** 10.1001/jamanetworkopen.2022.16910

**Published:** 2022-06-14

**Authors:** Douglas Barthold, Kai Yeung, David Lieberman, A. Mark Fendrick

**Affiliations:** 1 The Comparative Health Outcomes, Policy, and Economics (CHOICE) Institute, Department of Pharmacy, School of Pharmacy, University of Washington, Seattle; 2 Kaiser Permanente Washington Health Research Institute, Seattle; 3 Division of Gastroenterology and Hepatology, School of Medicine, Oregon Health Sciences University, Portland; 4 Center for Value-Based Insurance Design, University of Michigan, Ann Arbor, Michigan

## Abstract

This cohort study examines the colorectal cancer (CRC) screening rates in 2 US states that have implemented policies to eliminate consumer cost-sharing of CRC screening and compares these rates with those of neighboring states that have not enacted similar policies.

## Introduction

The Affordable Care Act requires that several colorectal cancer (CRC) screening modalities be covered with no cost-sharing for eligible individuals with average risk. However, cost barriers remain for some individuals with a positive test that requires a follow-up colonoscopy.^1^ In response, state-level policies have been enacted in Kentucky (2016), Oregon (2017), and California (2021) to eliminate financial disincentives that may deter follow-up colonoscopy for these individuals. In January 2022, federal guidance was issued to remove cost-sharing for colonoscopies following noninvasive CRC screening tests for commercial insurers, and a similar policy is under consideration for Medicare.^2^ We examined the CRC screening rates in Oregon and Kentucky and compared them with the rates of neighboring states without similar policies.

## Methods

This cohort study was deemed exempt from Institutional Review Board approval and informed consent owing to deidentified data. The study followed the STROBE reporting guideline.

We used repeated cross sections of the MarketScan Commercial Claims and Encounters Database from January 1, 2012, to December 31, 2019, to compare use of any CRC screening and receipt of a colonoscopy among individuals who received a CRC screening other than colonoscopy in Oregon vs Idaho and Washington and in Kentucky vs Indiana, Tennessee, and West Virginia using adjusted logistic regression. Analyses were adjusted for state fixed effects, year fixed effects, age, age squared, sex, and health care plan. The samples were restricted to person-years for individuals aged 45 to 64 years with 12 months of continuous enrollment in self-funded plans. Oregon and comparator states were further restricted to ages 50 to 64 years and excluded enrollees with high-deductible plans.

Statistical analyses were conducted using Stata, version 17.0 (StataCorp LLC); 2-sided *P* = .05 was considered significant. Data were analyzed between July 1, 2021, and January 31, 2022.

## Results

The sample constituted 2 327 935 person-years among 1 215 580 individuals (51.5% women; mean [SD] age, 54.5 [5.1] years). Details regarding CRC screening use are provided in Table 1. Individuals in Oregon had 6% higher odds of receiving any CRC screening after policy implementation (odds ratio [OR], 1.06 [95% CI, 1.00-1.06]; *P* = .03) compared with neighboring states that did not implement a similar policy (Table 2). Individuals receiving CRC screening in Oregon after policy implementation had 35% higher odds of undergoing an initial noninvasive test (OR, 0.65 [95% CI, 0.58-0.73]; *P* < .001) (Table 2). There were no significant differences in total CRC screening use in Kentucky after policy implementation compared with neighboring states (Table 2). Similarly, compared with neighboring states, the odds of receiving a colonoscopy conditional on undergoing noninvasive CRC screening were not statistically different in Oregon or Kentucky (Table 2).

**Table 1.** Use of CRC Screening in States With Policy Changes and Comparator States^a^

	Total	Oregon	Oregon comparator states^b^	Kentucky	Kentucky comparator states^c^
No. of person-years	2 327 935	112 672	497 606	392 642	1 325 015
Any CRC screening	342 554 (14.7)	15 659 (13.9)	68 880 (13.8)	58 191 (14.8)	199 824 (15.1)
Colonoscopy	190 063 (8.2)	11 096 (9.8)	46 102 (9.3)	33 077 (8.4)	99 788 (7.5)
NCS	181 443 (7.8)	5319 (4.7)	27 000 (5.4)	30 567 (7.8)	118 557 (8.9)
Colonoscopy (conditional on NCS)	28 952 (1.2)	756 (0.7)	4222 (0.8)	5453 (1.4)	18 521 (1.4)

Abbreviations: CRC, colorectal cancer; NCS, noncolonoscopy CRC screening.

^a^ Sample obtained from the MarketScan Commercial Claims and Encounters Database for the period 2012 to 2019. Data are presented as the number (%) of individuals unless indicated otherwise.

^b^ Idaho and Washington.

^c^ Indiana, Tennessee, and West Virginia.

**Table 2.** Adjusted Odds Ratios for CRC Screening in Oregon and Kentucky After Policy Implementation^a^

Screening type	Oregon		Kentucky	
	OR (95% CI)	*P* value	OR (95% CI)	*P* value
Any CRC screening	1.06 (1.00-1.06)	.03	1.00 (0.96-1.05)	>.99
Colonoscopy (among CRC screenings)	0.65 (0.58-0.73)	<.001	0.97 (0.89-1.07)	.57
Colonoscopy (conditional on NCS)	0.99 (0.78-1.27)	.96	1.01 (0.86-1.18)	.91

Abbreviations: CRC, colorectal cancer; NCS, noncolonoscopy colorectal cancer screening; OR, odds ratio.

^a^ Adjusted analyses compared Oregon (after 2017) with Idaho and Washington and compared Kentucky (after 2016) with Indiana, Tennessee, and West Virginia. Difference-in-differences logistic regression adjusted for state fixed effects, year fixed effects, age, age squared, sex, and health care plan (basic, comprehensive, exclusive provider organization; health maintenance organization; point of service; preferred provider organization; and point of service with capitation).

## Discussion

The Affordable Care Act eliminated cost-sharing for several CRC screening modalities. Because many individuals in the US undergo noninvasive CRC screening as an initial test, clinical guidelines state that a positive test requires a follow-up colonoscopy.^3^ However, several individuals who test positive on such tests incur out-of-pocket costs that may impede completion of the screening process or create financial hardship.^1^ Consequently, state-specific policies and recent federal guidance have eliminated cost-sharing for follow-up colonoscopy.

After comparing outcomes in 2 states that eliminated consumer cost-sharing for follow-up colonoscopy with those of neighboring states without such regulations, we found that access to full coverage significantly increased overall CRC screening and use of noninvasive testing in Oregon but not Kentucky. However, there was no significant increase in colonoscopies among individuals who received an initial noninvasive screening test in either state. Future studies should examine whether the use of noninvasive testing improves cost-effectiveness and reduces risk for patients.

Study limitations include an inability to observe screening test results in claims data and possible unmeasured confounding. Regardless, our results are consistent with previous research demonstrating an association between policies that eliminate out-of-pocket costs and use of cancer screening.^4^ These findings suggest that the enactment of policies that remove financial barriers is merely one of many elements (eg, health literacy, outreach, transportation, access to care) that may help to achieve desired cancer screening outcomes.
