# Error in Results and Table

**DOI:** 10.1001/jamanetworkopen.2022.24390

**Published:** 2022-07-12

In the Research Letter titled, “Comparison of Screening Colonoscopy Rates After Positive Noninvasive Testing for Colorectal Cancer in States With and Without Cost-Sharing,”^1^ published June 14, 2022, there was an error in the Results and in Table 2. The 95% CI for the odds ratio of patients in Oregon receiving any colorectal cancer screening after policy implementation should have been listed as 1.00 to 1.12. This article has been corrected.^1^

## References

[1] Barthold D, Yeung K, Lieberman D, Fendrick AM. Comparison of screening colonoscopy rates after positive noninvasive testing for colorectal cancer in states with and without cost-sharing. JAMA Netw Open. 2022;5(6):e2216910. doi:10.1001/jamanetworkopen.2022.16910 35699960 PMC9198727

